# HIV-related misinformation, testing, and non-disclosure in selected urban and peri-urban areas of West Nile, Eastern, and Northern Uganda

**DOI:** 10.1186/s41043-025-01134-4

**Published:** 2025-11-26

**Authors:** Doreen Nakalembe, Bridget Nagawa Tamale, Trinah Salome Kyomugisha, Aisha Nalugya, Jovan Galiwango, Joana Nakiggala, Patience Oputan, Junior Mike Wejuli, Joselyn Ndibalekera, John Bosco Isunju, Richard K. Mugambe, Tonny Ssekamatte, Justine Bukenya

**Affiliations:** 1 Makerere University School of Public Health, Makerere University, P.O. Box 7072, Kampala, Uganda; 2 SWEEM Health Consults Limited, P.O. Box 114574, Kampala, Uganda; 3 Ministry of Water and Environment, P.O. Box 20026, Kampala, Uganda

## Abstract

**Background:**

In Uganda, urban centres face a high prevalence of HIV, exacerbated by misinformation(inaccurate or false beliefs about HIV transmission, prevention, or treatment), low testing rates, and non-disclosure of HIV status (not revealing one’s HIV test result, particularly to sexual partners).However, evidence on the prevalence and determinants of HIV-related misinformation, testing, and disclosure within these urban and peri-urban centres remains limited. This study assessed the prevalence of HIV testing, misinformation, and non-disclosure in selected urban and peri-urban centres of West Nile, Eastern and Northern Uganda to inform targeted interventions aimed at improving HIV awareness, increasing the uptake of HIV testing services, and facilitating disclosure.

**Methods:**

A cross-sectional study was conducted among 930 households in five urban and peri-urban centres in Uganda. A multistage sampling technique was used to select participants. A digitised, structured questionnaire preloaded on the KoboCollect mobile application was used to collect data. Data were downloaded in Microsoft Excel and exported into Stata version 14 for statistical analysis. Modified Poisson regression was used to determine the factors associated with the outcome variables.

**Results:**

About 93.2% of respondents were misinformed about HIV, 20.8% had not disclosed their HIV status, and 3.3% had never been tested for HIV. Having a primary level of education (PR:0.96, 95% CI:0.93–0.99, *p* = 0.035) and spending more than 6 years in the area (PR: 1.05, 95% CI:1.00-1.10, *p* = 0.029) were associated with “HIV-related misinformation.” Being knowledgeable about some special drugs that a doctor or a nurse could give to a woman infected with the HIV/AIDs virus to reduce the risk of transmission to the baby (PR:1.03, 95% CI:1.00-1.07, *p* = 0.024) was associated with “HIV testing”. Being married (PR:0.89, 95% CI:0.65 − 0.12, *p* < 0.001 was associated with “non-disclosure” of HIV status.

**Conclusion:**

This study highlights the ongoing challenge of HIV/AIDS misinformation among urban populations in Uganda. While progress has been made in testing and disclosure, factors such as education, length of residence, and knowledge significantly influence these outcomes. These findings emphasise the importance of targeted educational interventions that provide clear, accurate HIV/AIDS information to enhance awareness, increase testing rates, and support the disclosure of HIV status.

## Background

 The HIV/AIDS pandemic remains a significant public health concern, having caused over 40 million deaths since the early 1980 s, when it was first clinically recognized [1, 2]. Despite major advances in HIV treatment, care, and prevention [3], the global burden remains substantial. By 2024, an estimated 40.8 million people were living with HIV worldwide, with 1.3 million new infections and 630,000 AIDS-related deaths [4]. Women and girls represent more than half of those living with HIV, and two-thirds of the global burden is concentrated in Africa [5]. Uganda reported its first HIV case in 1982 [6]. National prevalence is estimated at 5.8% among adults, 7.2% among women, and 4.3% among men [7]. Among adolescents aged 15–19 years, prevalence is 1.7% for girls and 0.2% for boys [7]. In contrast, prevalence is highest among adults aged 45–54 years, reaching 13.6% in women and 11.1% in men [7]. Importantly, HIV prevalence in urban areas (7.5%) exceeds that in rural areas (5.8%) and the general population (5.1%).

A combination of behavioral, socio-economic, and health system factors drives the elevated urban burden. High-risk sexual practices, including multiple partnerships, inconsistent condom use, and sex under the influence of psychoactive substances, are common in urban areas [8–12]. Rapid urbanization has also created vulnerabilities such as overcrowded housing, unemployment, and poor sanitation, which increase susceptibility to infection. Despite the high concentration of health facilities and programs in urban areas, access to HIV prevention and treatment remains constrained by stigma, financial barriers, long waiting times, inadequate youth-friendly services, and commodity stockouts [13, 14]. Notably, this paradox exists despite urban residents having higher education levels, greater exposure to information and technology, and closer proximity to health services than their rural counterparts.

Beyond these behavioral and structural risks, HIV transmission in urban settings is further complicated by widespread misinformation. Misconceptions about HIV prevention and treatment persist despite decades of education and awareness campaigns [15–17]. In this study, HIV-related misinformation refers to inaccurate beliefs, such as the idea that herbal remedies can cure HIV or that HIV can be transmitted through mosquito bites [17–20]. According to the 2022 Uganda Demographic and Health Survey (UDHS), nearly half of Ugandans still lack comprehensive knowledge about HIV/AIDS [21]. Such misinformation perpetuates stigma, discourages testing and disclosure, and undermines adherence to antiretroviral therapy (ART) [17–20]. HIV testing and disclosure are critical for reducing transmission, yet remain suboptimal in Uganda. While approximately 90% of people living with HIV know their status, approaching the first 95 target of the UNAIDS 95-95-95 goals [22], disclosure of HIV serostatus to sexual partners is still hindered by fear of stigma and discrimination [23, 24]. Furthermore, misinformation directly influences disclosure practices by reinforcing misconceptions and mistrust between partners [25, 26].

To address these challenges, global and national initiatives have prioritized HIV education, testing, and disclosure. For example, UNAIDS and WHO support campaigns such as World AIDS Day to raise awareness and dispel myths [27]. In Uganda, the third National Development Plan (2020/21–2024/25), the Presidential Fast-Track Initiative on Ending AIDS by 2030, and the Presidential Initiative on AIDS Strategy for Communication to Youth (PIASCY) have been launched to improve prevention and testing [27–29]. Despite these extensive efforts, HIV prevalence remains disproportionately high in urban populations, where misinformation spreads rapidly through digital and social networks [7]. This study, therefore assessed the extent of HIV-related misinformation, testing, and disclosure among urban dwellers in Uganda to inform more targeted, culturally sensitive interventions.

## Materials and methods

### Study design and setting

We applied a cross-sectional study design to obtain data from participants in five urban centres in Uganda between April and May 2022. This study was conducted in the town councils of Namasale, Ngora, and Nyero and the municipalities of Koboko and Kumi. Kumi municipality, Ngora and Nyero town councils are situated in the Eastern part of Uganda, close to Soroti and Mbale Cities [30]. Kumi district, the home of Kumi Municipality and Nyero town council, has 35 healthcare facilities (HCFs), of which 3 are clinics, 17 are HCIIs, 8 are HCIIIs, 3 are HCIVs and 4 are hospitals [31]. Ngora district, the home of Ngora town council, has 15 HCFs, of which 7 are HCIIs, 6 are HCIIIs, 1 HCIV, and one hospital [31]. Conversely, Koboko District, where Koboko Municipality lies, is located in the West Nile Region of Uganda, approximately 55 km away from Arua city [32]. The district has 21 HCFs, comprising four clinics, 9 HCIIs, 7 HCIIIs and one hospital [31]. Namasale Town Council in Northern Uganda is roughly 154 km and 143 km from Soroti and Lira Cities, respectively [33]. Amolatar district, where Namasale town council is located, has 15 HCFs, of which 2 are clinics, 8 are HCIIs, 3 are HCIIIs, 1 HCIV and one hospital [31]. The selection of these urban centres was based on geographical diversity, thus representing the different regions in Uganda. Namasale Town Council, for example, is a landing site where a considerable proportion of the population is engaged in fishing [34] and is not often available to participate in research activities. Furthermore, most urban respondents are often too busy and fatigued to participate in interviews. In addition, Koboko Municipality is a border town where some individuals engage in cross-border trade [35], limiting their participation.

### Study population, eligibility criteria, sample size estimation, and sampling

The study population included householders or their next of kin. The study included individuals aged 18 years and above who had been residents for at least six months and excluded those who were not feeling well or had any sickness during the study. “Sick” in this context referred to individuals with any acute or severe illness that could hinder their ability to provide reliable responses during the survey. This included, but was not limited to, conditions such as high fever, difficulty breathing, or visible signs of severe discomfort. The determination of sickness was based on participant self-reporting or visible observation by the research team during recruitment. We used the Kish-Leslie formula for cross-sectional studies to estimate the required sample. A proportion of non-disclosure of HIV status of 50.0% (due to limited literature on non-disclosure of HIV status in urban settings), a z-score of 1.96 corresponding to the 95% level of confidence, and a margin of error of 5% was used to estimate a minimum sample size of 385 respondents. Factoring in a 2.0 design effect due to variance distortion between urban centres [36, 37] and a non-response rate of 20% [38, 39], obtaining a minimum sample size of **924**. The non-response rate was informed by previous literature [38, 39] and experience conducting surveys in urban areas. However, 930 respondents were included in the final analysis due to fieldwork adjustments.

**Fig. 1:**
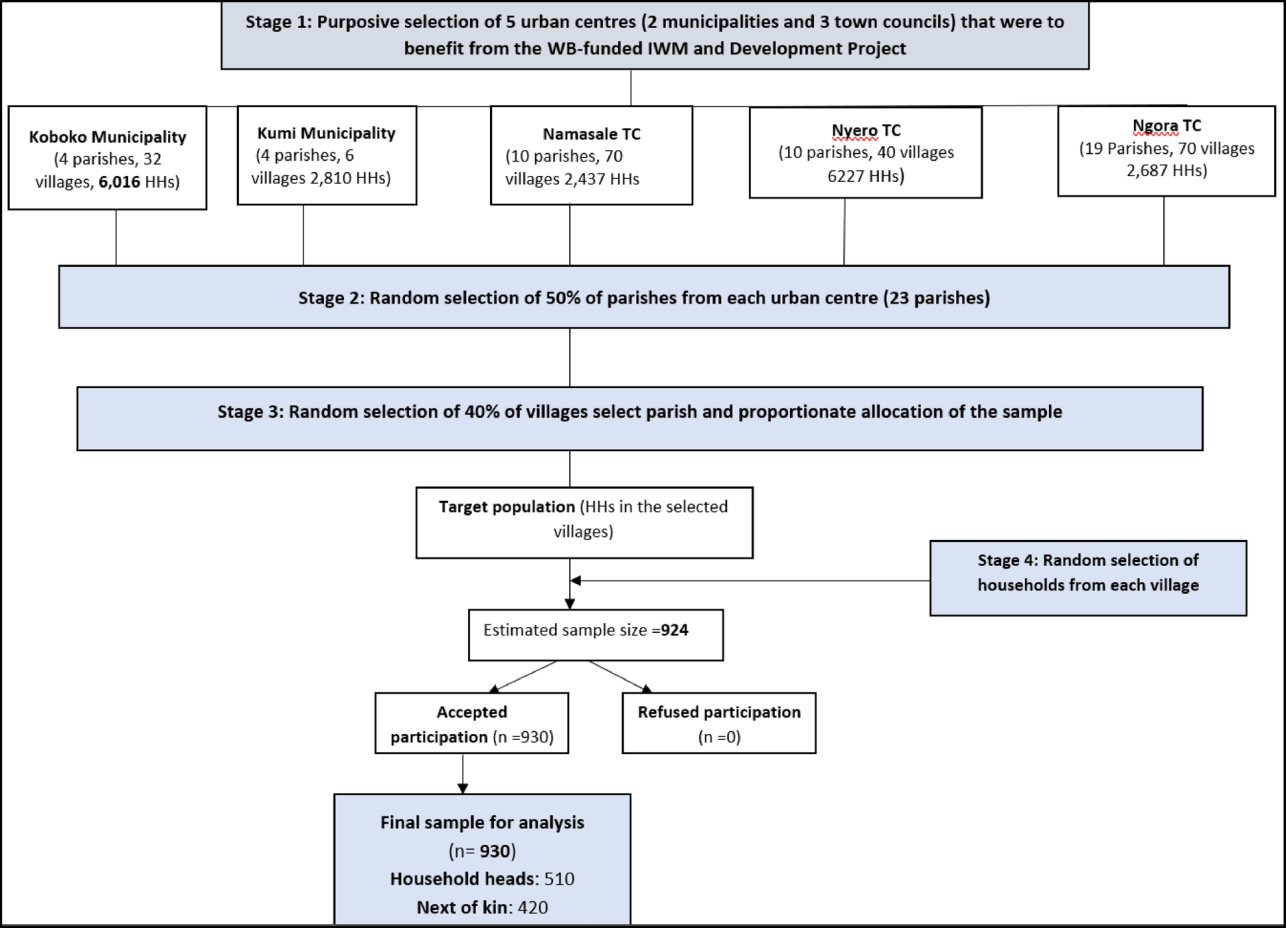
A flowchart showing the four-stage sampling process

We used a four-stage multistage sampling technique to select respondents. The first stage involved the purposive selection of five urban centres that would benefit from the World Bank-funded Integrated Water Management and Development Project. As a risk mitigation measure, the project required a rapid assessment of HIV-related knowledge and practices and gender-based violence. The second stage involved randomly selecting 50% of the parishes in each urban centre. We obtained a list of the cells/villages and households in the selected divisions from the local council leadership. In the third stage, we then randomly selected 40% of the cells/villages, followed by a random selection of households using the Microsoft Excel randomiser during the fourth stage. At the household level, the research assistants purposively selected the householders, and, in their absence, the next of kin were interviewed (Fig. 1 and Table 1).

**Table 1:** Sample size distribution

Township	Total of households	Estimated proportionate sample
Koboko Municipality	6016	277
Kumi Municipality	2810	130
Namasale Town council	2437	112
Nyero town	6227	287
Ngora Town council	2687	124
Total	88,876	930

### Data collection procedures, quality assurance, and control

The study utilised an electronic structured questionnaire created using KoboCollect software version 2021, 2.4 [40]. The questionnaire included skips and validation criteria to minimise errors and incorporated standardised questions from the Uganda Demographic Health Survey (UDHS) [41]. The Kobo toolbox server was used to design the questionnaire, which was later uploaded to the mobile application pre-installed on Android-enabled smart devices. The Kobo Collect data entry software was designed with skips and restrictions to eliminate missing data and inconsistencies. All questions were mandatory to reduce missing data. Participants who chose not to answer a specific question were provided with the option to select “declined to answer.” All research assistants underwent a two-day training to familiarise themselves with the data collection tools, ethical principles, and the study protocol. The electronic questionnaire was pretested in Mukono municipality to enhance the clarity of the questions, the validity of the online tool, and to determine the interview duration. Mukono municipality was selected due to its similar characteristics to the selected urban centres where the study was conducted. Research assistants received thorough supervision to ensure adherence to the study protocol and ethical guidelines. While in the field, research assistants were required to upload data to the cloud server daily for quality control purposes. Debriefing sessions were conducted at the end of each field day to address any challenges encountered. Data were uploaded to the server daily and checked for completeness by the data manager. Data were secured on the cloud server, which was password-protected to prevent unauthorised access. Only the research team had access to this data. All data were encrypted and stored on a secure, password-protected server to ensure data security and confidentiality. Access was restricted to authorised study personnel, and personally identifiable information was not collected to protect respondent privacy. Data transmission was secured using end-to-end encryption, and backups were conducted regularly to prevent data loss.

### Study variables and measurement

#### Outcome variables

The outcome variables included HIV-related misinformation, ever testing for HIV, and disclosure of HIV status.

##### HIV-related misinformation

We used a total of 15 questions to establish HIV-related misinformation about HIV/AIDS. Respondents were asked if a man/woman had HIV, would his/her partner also have HIV (Yes = 0, No = 1), if people could get HIV infection or HIV/AIDS from mosquito bites (Yes = 0, No = 1), if people could get HIV by sharing food with a person who had AIDs (Yes = 0, No = 1), if people could get HIV because of witchcraft or other supernatural means (Yes = 0, No = 1), if HIV was treated by prayer (Yes = 0, No = 1), if HIV was treated by herbal medicine (Yes = 0, No = 1), if it was possible for a healthy-looking person to have HIV (Yes = 1, No = 0), if HIV was transmitted from a mother to her baby during pregnancy (Yes = 1, No = 0), if HIV was transmitted from a mother to her baby during delivery (Yes = 1, No = 0), if HIV was transmitted from a mother to her baby during breastfeeding (Yes = 1, No = 0), if people reduced their chances of getting HIV by having just one uninfected sex partner who had no other sex partners (Yes = 1, No = 0), if people reduced their chance of getting HIV by using a condom every time they had sex (Yes = 1, No = 0), if someone prevented her/himself from acquiring HIV/AIDS through abstinence (Yes = 1, No = 0), if safe male circumcision helped to reduce the risk of acquiring HIV/AIDs (Yes = 1, No = 0), and if there were any special drugs that a doctor or a nurse could give to a woman infected with HIV to reduce the risk of transmission to the baby (Yes = 1, No = 0).

The total score for HIV-related misinformation for each respondent was obtained by adding up scores obtained from each question. Respondents who had a total score of 15 were considered informed, while those who scored between 0 and 14 were misinformed.

##### HIV testing and non-disclosure of the HIV status

The prevalence of HIV testing was established by asking respondents if they had ever tested for HIV. The variable was coded “1” for respondents who had ever tested and “0” for those who had never. Non-disclosure of HIV status was measured using self-reports. Respondents who had ever tested for HIV and reported having a recent sexual partner were asked if they had ever disclosed their status to that partner. Those who responded “No” were coded as “1”, and those who responded “Yes” were coded as “0”.

### Independent variables

The independent variables included age, sex, highest level of education, religion, marital status, years spent in the area, type of housing, employment status, and perceived social status. Perceived social status was assessed using the McArthur Scale of Subjective Social Status [42]. Respondents were presented with a diagram of a ladder with 10 rungs representing the socio-economic spectrum, from those best off at the top to those worst off at the bottom. Participants selected the rung that they believed represented their position relative to others in their community. Based on their responses, social status was categorised as follows: individuals placing themselves on the upper rungs (7–10) were categorised as having high social status, those on the middle rungs (4–6) were considered to have medium social status, and those on the lower rungs (1–3) were classified as having low social status [42].

### Data management and statistical analyses

Data were downloaded as a Microsoft Excel file, cleaned, and later exported to STATA version 14.0 for statistical analysis. Descriptive statistics were performed to summarise the variables. Frequencies and percentages were used to summarise the prevalence of HIV-related misinformation, testing, and disclosure. Data were stratified by sex and age category of the respondent. Stratifying by age and sex helps to create a representative sample that accurately reflects the population’s diversity, allowing for more precise and meaningful analyses [43]. In addition, continuous variables like age were summarised using mean and standard deviation.

The prevalence of the outcomes of interest (HIV misinformation, testing, and non-disclosure) was above 10% [44]. Therefore, a modified Poisson regression model was used to determine the factors associated with those variables. Bivariate analysis was performed to examine the relationship between the predictors and dependent variables. For multivariable analysis, we used stepwise backward elimination to identify the best-fitting model. All variables with a p-value < 0.25 in the bivariate analysis were considered potential predictors and were included in the initial complete model. The inclusion of variables with a p-value less than 0.25 ensures a broader range of variables in the analysis, which allows for a more extensive adjustment for potential confounders [45]. Even though the age and sex of the respondents were not significant during the bivariate analysis, they were included in the multivariable analysis to control for confounding [46]. We then used stepwise backward selection, sequentially removing non-significant variables based on the Akaike Information Criterion (AIC) until only statistically significant predictors remained in the final model (*p* < 0.05). A backward elimination rule starts with all possible explanatory variables and then discards the least statistically significant variables, one by one. The discarding stops when each variable remaining in the equation is statistically significant [47]. During this process, the least significant variables, indicated by the highest p-values, were systematically removed one by one. This step-by-step reduction continued until only the most statistically significant variables remained, which were less than 0.05 at a 95% level of confidence, which ensured that the final model was parsimonious and robust.

## Results

### Socio-demographic characteristics

A total of 930 respondents were interviewed. Of whom, 74.0% (688/930) were females, 77.4% (720/930) were either married or cohabiting, 30.8% (286/930) were Roman Catholics, and 38.9% (362/930) had attained primary as their highest level of education. A higher proportion of females, 63.1% (434/688), compared to the males, 52.9% (128/242), had a low perceived social status. A lower proportion of respondents aged 25 years and above, 16.2% (135/831), compared to those aged between 18 and 24 years, 18.2% (18/99), had semi-permanent housing (Table 2).

**Table 2:** Socio-demographic characteristics among respondents

Variable	Category	Overall (*N* = 930)	Sex of respondent		Age-category (Years)	
			Female (*n* = 688)	Male (*n* = 242)	18–24 (*n* = 99)	25 and above (*n* = 831)
Marital status	Divorced/Separated	82 (8.8)	78 (11.3)	4 (1.7)	3 (3.0)	79 (9.5)
	Married/Cohabiting	720 (77.4)	504 (73.3)	216 (89.3)	65 (65.7)	655 (78.8)
	never married/Single	63 (6.8)	45 (6.5)	18 (7.4)	31 (31.3)	32 (3.9)
	Widow/Widower	65 (7.0)	61 (8.9)	4 (1.7)	0 (0.0)	65 (7.8)
Religion	Anglican	405 (43.5)	297 (43.2)	108 (44.6)	48 (48.5)	357 (43.0)
	Muslim	107 (11.5)	85 (12.4)	22 (9.1)	13 (13.1)	94 (11.3)
	Other	12 (1.3)	10 (1.5)	0 (0.0)	0 (0.0)	11 (1.3)
	Pentecost/Born again	120 (12.9)	92 (13.4)	28 (11.6)	7 (7.1)	113 (13.6)
	Roman Catholic	286 (30.8)	204 (29.7)	82 (33.9)	30 (30.3)	256 (30.8)
Highest level of education	No formal education	85 (9.1)	78 (11.3)	7 (2.9)	3 (3.0)	82 (9.9)
	Primary	362 (38.9)	299 (43.5)	63 (26.0)	38 (38.4)	324 (39.0)
	Secondary	301 (32.4)	199 (28.9)	102 (42.1)	49 (49.5)	252 (30.3)
	Tertiary	182 (19.6)	112 (16.3)	70 (28.9)	9 (9.1)	173 (20.8)
Perceived social status	Low	562 (60.4)	434 (63.1)	128 (52.9)	62 (62.6)	500 (60.2)
	Middle	339 (36.5)	234 (34.0)	105 (43.4)	33 (33.3)	306 (36.8)
	High	29 (3.1)	20 (2.9)	9 (3.7)	4 (4.0)	25 (3.0)
Type of housing	Permanent	609 (65.5)	459 (66.7)	150 (62.0)	60 (60.6)	549 (66.1)
	Semi-permanent	153 (16.5)	103 (15.0)	50 (20.7)	18 (18.2)	135 (16.2)
	Temporary	168 (18.1)	126 (18.3)	42 (17.4)	21 (21.2)	147 (17.7)
Years spent in the area	1–5 years	258 (27.7)	219 (31.8)	39 (16.1)	51 (51.5)	207 (24.9)
	6–10 years	86 (9.2)	71 (10.3)	15 (6.2)	9 (9.1)	77 (9.3)
	Above 10 years	586 (63.0)	398 (57.8)	188 (77.7)	39 (39.4)	547 (65.8)

### Awareness of HIV/AIDS

Nearly all, 93.2% (867/930) (95% CI: 91.0–94.0) of the respondents were misinformed about HIV. A higher proportion of females, 57.0% (392/688), compared to males, 46.3% (112/242), mentioned that a man/woman always had HIV if the counterpart was also infected. A higher proportion of females, 10.2% (70/688), compared to males, 5.8% (14/242), mentioned that people could get HIV infection or HIV/AIDS from mosquito bites. A higher proportion of males, 13.6% (33/242), compared to females, 6.7% (46/688), mentioned that HIV could not be transmitted from a mother to her baby during delivery. A lower proportion of males, 21.5% (52/242), compared to females, 11.8% (81/688), mentioned that HIV could not be transmitted from a mother to her baby during breastfeeding. About 63.6% (63/99) of the respondents aged between 18 and 24 years mentioned that they would want to keep it a secret if a member of the family got infected with the HIV/AIDS virus. About 12.6% (105/831) of the respondents aged 25 years and above mentioned that a wife was not justified in asking her husband to use a condom when they had sex if she knew that she could get a disease during sexual intercourse. About 90.5% (842/930) of the respondents mentioned that people can reduce their chances of getting the HIV/AIDS virus by using a condom every time they have sex. About 95.7% (890/930) of the respondents mentioned that someone can prevent him/herself from acquiring HIV/AIDS through abstinence. More than a quarter, 28.5% (265/930) of the respondents, mentioned that male circumcision cannot help to reduce the risk of acquiring HIV/AIDS. About 30.5% (284/930) of the respondents mentioned that there were no special drugs that a doctor or a nurse could give to a woman infected with the HIV/AIDs virus to reduce the risk of transmission to the baby. About 12.3% (114/930) of the respondents mentioned that they would not buy fresh vegetables from a shopkeeper or vendor if they knew that the person had the HIV/AIDs virus. About 8.0% (74/930) of the respondents mentioned that they would not be willing to care for a family member in case he/she became sick within their household (Table 3).

**Table 3:** Awareness of HIV/AIDS in selected urban and peri-urban areas of West Nile, Eastern, and Northern Uganda

Variable	Category	Overall (*N* = 930)	Sex of respondent		Age-category (years)	
			Female (*n* = 688)	Male (*n* = 242)	18–24 (*n* = 99)	25 and above (*n* = 831)
A man/woman always has HIV if the counterpart is also infected	No	426 (45.8)	296 (43.0)	130 (53.7)	44 (44.4)	382 (46.0)
	Yes	504 (54.2)	392 (57.0)	112 (46.3)	55 (55.6)	449 (54.0)
It is possible for a healthy-looking person to have HIV	No	113 (12.2)	83 (12.1)	30 (12.4)	21 (21.2)	92 (11.1)
	Yes	817 (87.8)	605 (87.9)	212 (87.6)	78 (78.8)	739 (88.9)
Knows of a place(s) where people can get tested for HIV	No	32 (3.4)	24 (3.5)	8 (3.3)	9 (9.1)	23 (2.8)
	Yes	898 (96.6)	664 (96.5)	234 (96.7)	90 (90.9)	808 (97.2)
People can get an HIV infection or HIV/AIDS from mosquito bites	No	846 (91.0)	618 (89.8)	228 (94.2)	90 (90.9)	756 (91.0)
	Yes	84 (9.0)	70 (10.2)	14 (5.8)	9 (9.1)	75 (9.0)
People can get the HIV/AIDS virus by sharing food with a person who has AIDS	No	893 (96.0)	657 (95.5)	236 (97.5)	95 (96.0)	798 (96.0)
	Yes	37 (4.0)	31 (4.5)	6 (2.5)	4 (4.0)	33 (4.0)
People can get the HIV/AIDS virus because of witchcraft or other supernatural means	No	881 (94.7)	656 (95.3)	225 (93.0)	95 (96.0)	786 (94.6)
	Yes	49 (5.3)	32 (4.7)	17 (7.0)	4 (4.0)	45 (5.4)
HIV can be transmitted from a mother to her baby during pregnancy	No	458 (49.2)	327 (47.5)	131 (54.1)	54 (54.5)	404 (48.6)
	Yes	472 (50.8)	361 (52.5)	111 (45.9)	45 (45.5)	427 (51.4)
HIV can be transmitted from a mother to her baby during delivery	No	79 (8.5)	46 (6.7)	33 (13.6)	14 (14.1)	65 (7.8)
	Yes	851 (91.5)	642 (93.3)	209 (86.4)	85 (85.9)	766 (92.2)
HIV can be transmitted from a mother to her baby during breastfeeding	No	133 (14.3)	81 (11.8)	52 (21.5)	20 (20.2)	113 (13.6)
	Yes	797 (85.7)	607 (88.2)	190 (78.5)	79 (79.8)	718 (86.4)
People can reduce their chances of getting the HIV virus by having just one uninfected sex partner who has no other sex partners	No	81 (8.7)	66 (9.6)	15 (6.2)	10 (10.1)	71 (8.5)
	Yes	849 (91.3)	622 (90.4)	227 (93.8)	89 (89.9)	760 (91.5)
People can reduce their chances of getting the HIV/AIDS virus by using a condom every time they have sex	No	88 (9.5)	71 (10.3)	17 (7.0)	10 (10.1)	78 (9.4)
	Yes	842 (90.5)	617 (89.7)	225 (93.0)	89 (89.9)	753 (90.6)
Someone can prevent her/himself from acquiring HIV/AIDS through abstinence	No	40 (4.3)	33 (4.8)	7 (2.9)	6 (6.1)	34 (4.1)
	Yes	890 (95.7)	655 (95.2)	235 (97.1)	93 (93.9)	797 (95.9)
Male circumcision can help to reduce the risk of acquiring HIV/AIDS	No	265 (28.5)	200 (29.1)	65 (26.9)	29 (29.3)	236 (28.4)
	Yes	665 (71.5)	488 (70.9)	177 (73.1)	70 (70.7)	595 (71.6)
There are some special drugs that a doctor or a nurse can give to a woman infected with the HIV/AIDS virus to reduce the risk of transmission to the baby	No	284 (30.5)	196 (28.5)	88 (36.4)	34 (34.3)	250 (30.1)
	Yes	646 (69.5)	492 (71.5)	154 (63.6)	65 (65.7)	581 (69.9)
Would buy fresh vegetables from a shopkeeper or vendor if you knew that this person had the HIV/AIDS virus	No	114 (12.3)	92 (13.4)	22 (9.1)	18 (18.2)	96 (11.6)
	Yes	816 (87.7)	596 (86.6)	220 (90.9)	81 (81.8)	735 (88.4)
Would want to keep it a secret if a member of the family got infected with the HIV/AIDS virus	No	357 (38.4)	249 (36.2)	108 (44.6)	36 (36.4)	321 (38.6)
	Yes	573 (61.6)	439 (63.8)	134 (55.4)	63 (63.6)	510 (61.4)
Would be willing to care for the family member in case he/she became sick within the respondent’s household	No	74 (8.0)	65 (9.4)	9 (3.7)	6 (6.1)	68 (8.2)
	Yes	856 (92.0)	623 (90.6)	233 (96.3)	93 (93.9)	763 (91.8)
A female teacher should be allowed to continue teaching in school if she has the HIV/AIDS virus but is not sick	No	100 (10.8)	84 (12.2)	16 (6.6)	9 (9.1)	91 (11.0)
	Yes	830 (89.2)	604 (87.8)	226 (93.4)	90 (90.9)	740 (89.0)
Children aged 10–14 years should be taught about using a condom to avoid getting HIV/AIDS	No	480 (51.6)	371 (53.9)	109 (45.0)	58 (58.6)	422 (50.8)
	Yes	450 (48.4)	317 (46.1)	133 (55.0)	41 (41.4)	409 (49.2)
Has ever heard about other infections that can be transmitted through sexual contact apart from AIDS	No	100 (10.8)	82 (11.9)	18 (7.4)	18 (18.2)	82 (9.9)
	Yes	830 (89.2)	606 (88.1)	224 (92.6)	81 (81.8)	749 (90.1)
A wife is justified in asking her husband to use a condom when they have sex if she knows that she can get a disease during sexual intercourse	No	123 (13.2)	94 (13.7)	29 (12.0)	18 (18.2)	105 (12.6)
	Yes	807 (86.8)	594 (86.3)	213 (88.0)	81 (81.8)	726 (87.4)
A wife is justified in refusing to have sex with her husband when she knows he has sex with other women	No	220 (23.7)	162 (23.5)	58 (24.0)	36 (36.4)	184 (22.1)
	Yes	710 (76.3)	526 (76.5)	184 (76.0)	63 (63.6)	647 (77.9)
Can you say no to your (husband/partner) if you do not want to have sexual intercourse	No	207 (22.3)	166 (24.1)	41 (16.9)	26 (26.3)	181 (21.8)
	Yes	723 (77.7)	522 (75.9)	201 (83.1)	73 (73.7)	650 (78.2)
Can you ask your (husband/partner) to use a condom if you want him to	No	236 (25.4)	185 (26.9)	51 (21.1)	39 (39.4)	197 (23.7)
	Yes	694 (74.6)	503 (73.1)	191 (78.9)	60 (60.6)	634 (76.3)
HIV can be treated by prayer	No	838 (90.1)	615 (89.4)	223 (92.1)	89 (89.9)	749 (90.1)
	Yes	92 (9.9)	73 (10.6)	19 (7.9)	10 (10.1)	82 (9.9)
HIV can be treated by herbal medicine	No	855 (91.9)	629 (91.4)	226 (93.4)	84 (84.8)	771 (92.8)
	Yes	75 (8.1)	59 (8.6)	16 (6.6)	15 (15.2)	60 (7.2)

### HIV testing and disclosure

Almost all, 96.7% (899/930) of the respondents had ever been tested for HIV. Of these, 23.4% (211/899) reported testing less than 3 months ago, 3.7% (33/899) had positive results from the HIV test, 78.5% (706/899) had disclosed their status to their sexual partners, and 67.0% (602/899) knew their partner’s HIV status (Table 4).

**Table 4:** HIV testing and care among respondents in selected urban and peri-urban areas of West Nile, Eastern, and Northern Uganda

Variable	Category	Overall (*N* = 930)	Sex of respondent		Age category (Years)	
			Female (*n* = 688)	Male (*n* = 242)	18–24 years (*n* = 99)	25 years and above (*n* = 831)
Has ever been tested for HIV	No	31 (3.3)	24 (3.5)	7 (2.9)	6 (6.1)	25 (3.0)
	Yes	899 (96.7)	664 (96.5)	235 (97.1)	93 (93.9)	806 (97.0)
Duration since last HIV test (*n* = 899)	3–5 months	155 (17.2)	120 (18.1)	35 (14.9)	18 (19.4)	137 (17.0)
	Less than 3 months	211 (23.5)	154 (23.2)	57 (24.3)	30 (32.3)	181 (22.5)
	6–11 months	167 (18.6)	132 (19.9)	35 (14.9)	21 (22.6)	146 (18.1)
	1–2 years	191 (21.2)	141 (21.2)	50 (21.3)	16 (17.2)	175 (21.7)
	More than 2 years	147 (16.4)	95 (14.3)	52 (22.1)	3 (3.2)	144 (17.9)
	Don’t know	28 (3.1)	22 (3.3)	6 (2.6)	5 (5.4)	23 (2.9)
HIV test result (*n* = 899)	Negative	831 (92.4)	616 (92.8)	215 (91.5)	88 (94.6)	743 (92.2)
	No response	35 (3.9)	22 (3.3)	13 (5.5)	4 (4.3)	31 (3.8)
	Positive	33 (3.7)	26 (3.9)	7 (3.0)	1 (1.1)	32 (4.0)
Disclosed HIV status to the sexual partner (*n* = 899)	No	193 (21.5)	161 (24.2)	32 (13.6)	27 (29.0)	166 (20.6)
	Yes	706 (78.5)	503 (75.8)	203 (86.4)	66 (71.0)	640 (79.4)
Knows partner’s status (*n* = 899)	No	297 (33.0)	250 (37.7)	47 (20.0)	38 (40.9)	259 (32.1)
	Yes	602 (67.0)	414 (62.3)	188 (80.0)	55 (59.1)	547 (67.9)
Has registered in a clinic for HIV care if the HIV status is positive (*n* = 33)	No	1 (3.0)	1 (3.8)	0 (0.0)	0 (0.0)	1 (3.1)
	Yes	32 (97.0)	25 (96.2)	7 (100.0)	1 (100.0)	31 (96.9)
He is currently on antiretroviral therapy (*n* = 33)	No	1 (3.0)	1 (3.8)	0 (0.0)	0 (0.0)	1 (3.1)
	Yes	32 (97.0)	25 (96.2)	7 (100.0)	1 (100.0)	31 (96.9)

### Socio-demographic factors associated with HIV-related misinformation in urban and peri-urban centres in Uganda

After adjusting for age and sex as potential confounders, only the highest level of education, years spent in the area, ever tested for HIV/AIDS, and employment status were significantly associated with HIV-related misinformation. The prevalence of HIV-related misinformation was 4% lower among respondents with a primary level of education compared to those with no formal education (PR:0.96, 95%CI:0.93–0.99, *p* = 0.035). The prevalence of HIV-related misinformation was 9% lower among respondents with a secondary level of education compared to those with none (PR:0.91, 95%CI:0.87–0.95, *p* < 0.001). Respondents who had spent more than 6 years in the area had a 5% higher prevalence of HIV-related misinformation compared to those who had spent between 1 and 5 years (PR: 1.05, 95% CI:1.00–1.10.00.10, *p* = 0.029). The prevalence of HIV-related misinformation was 6% lower among respondents who had ever tested for HIV compared to those who had not (PR:0.94, 95%CI:0.93–0.96, *p* < 0.001). The prevalence of HIV-related misinformation was 6% higher among unemployed respondents compared to those who were employed (PR: 1.06, 95% CI:1.01–1.11, *p* = 0.010) (Table 5).

**Table 5:** Socio-demographic factors associated with HIV-related misinformation in urban and peri-urban centers in Uganda

Variable	Category	n	Misinformation on HIV		Crude prevalence Ratio (95% CI)	Adjusted prevalence Ratio (95% CI)	p-value
			Informed (*n* = 63)	Misinformed (*n* = 867)			
Sex	Female	688	47 (6.8)	641 (93.2)	1	1	
	Male	242	16 (6.6)	226 (93.4)	1.00 (0.96–1.04)	1.01 (0.97–1.05)	0.522
Age-category (years)	18–24	99	5 (5.1)	94 (94.9)	1	1	
	≥ 25	831	58 (7.0)	773 (93.0)	0.97 (0.93–1.02)	0.96 (0.91–1.01)	0.149
Marital status	Divorced/Separated	82	5 (6.1)	77 (93.9)	1		
	Married/Cohabiting	720	51 (7.1)	669 (92.9)	0.98 (0.93–1.04)		
	Unmarried/Single	63	4 (6.3)	59 (93.7)	0.99 (0.91–1.08)		
	Widow/Widower	65	3 (4.6)	62 (95.4)	1.01 (0.94–1.09)		
Highest level of education	No formal education	85	1 (1.2)	84 (98.8)	1	1	
	Primary	362	16 (4.4)	346 (95.6)	0.96 (0.93–0.99)	0.96 (0.93–0.99)	0.035*
	Secondary	483	46 (73.0)	437 (50.4)	0.91 (0.88–0.95)	0.91 (0.87–0.95)	**p** < 0.001*
Years spent in the area	1–5 years	258	26 (10.1)	232 (89.9)	1	1	
	6 years and above	672	37 (5.5)	635 (94.5)	1.05 (1.00–1.09.00.09)	1.05 (1.00–1.10.00.10)	0.029*
Perceived social status	Low	562	36 (6.4)	526 (93.6)	1		
	Middle	339	25 (7.4)	314 (92.6)	0.98 (0.95–1.02)		
	High	29	2 (6.9)	27 (93.1)	0.99 (0.89–1.10)		
Type of housing	Permanent	609	50 (8.2)	559 (91.8)	1	1	
	Semi-permanent	153	6 (3.9)	147 (96.1)	1.04 (1.00–1.08.00.08)	1.03 (0.99–1.07)	0.140
	Temporary	168	7 (4.2)	161 (95.8)	1.04 (1.00–1.08.00.08)	1.01 (0.97–1.05)	0.460
Ever been tested for HIV	No	31	0 (0.0)	31 (100.0)	1	1	
	Yes	899	63 (7.0)	836 (93.0)	0.92 (0.91–0.94)	0.94 (0.93–0.96)	p < 0.001*
Employment status	Employed	878	62 (7.1)	816 (92.9)	1	1	
	Unemployment	52	1 (1.9)	51 (98.1)	1.05 (1.01–1.10)	1.06 (1.01–1.11)	0.010*

### Predictors of HIV testing

After adjusting for age and sex as potential confounders, only the presence of some special drugs that a doctor or a nurse could give to a woman infected with the HIV/AIDS Virus to reduce the risk of transmission to the baby was significantly associated with HIV testing. Respondents who knew that there were some special drugs that a doctor or a nurse could give to a woman infected with the HIV/AIDS virus to reduce the risk of transmission to the baby had a 3.0% (PR:1.03, 95% CI:1.00–1.07.00.07, *p* = 0.024) higher prevalence of getting HIV tested compared to those who didn’t know (Table 6). However, as the lower confidence interval boundary is 1.00, this finding should be interpreted with caution, as the observed association may be marginal or due to chance.

**Table 6:** Predictors of HIV testing among respondents in urban and peri-urban centers in Uganda

Variable	Category	n	Has ever tested for HIV		Crude prevalence ratio (CI = 95%)	Adjusted prevalence ratio (CI = 95%	P-value
			No (*n* = 31)	Yes (*n* = 899)			
Sex	Female	688	24 (3.5)	664 (96.5)	1	1	
	Male	242	7 (2.9)	235 (97.1)	1.00 (0.98–1.03)	0.99 (0.97–1.02)	0.996
Age-category (Years)	18–24	99	6 (6.1)	93 (93.9)	1	1	
	25 and above	831	25 (3.0)	806 (97.0)	1.03 (0.98–1.08)	1.02 (0.98–1.06)	0.227
Marital status	Divorced/Separated	82	2 (2.4)	80 (97.6)	1	1	
	Married/Cohabiting	720	17 (2.4)	703 (97.6)	1.00 (0.96–1.03)	1.00 (0.97–1.04)	0.753
	Unmarried/Single	63	5 (7.9)	58 (92.1)	0.94 (0.87–1.02)	0.96 (0.89–1.02)	0.249
	Widow/Widower	65	7 (10.8)	58 (89.2)	0.91 (0.83–1.00.83.00)	0.91 (0.84–1.00.84.00)	0.067
Perceived social status	Low	562	16 (2.8)	546 (97.2)	1		
	Middle	339	13 (3.8)	326 (96.2)	0.98 (0.96–1.01)		
	High	29	2 (6.9)	27 (93.1)	0.95 (0.86–1.05)		
Employment status	Employed	878	28 (3.2)	850 (96.8)	1		
	Unemployment	52	3 (5.8)	49 (94.2)	0.97 (0.90–1.04)		
It is possible to prevent victims of rape from contracting HIV even when raped by an HIV-positive person	Don’t know	85	6 (7.1)	79 (92.9)	1	1	
	False	241	9 (3.7)	232 (96.3)	1.03 (0.97–1.10)	1.02 (0.96–1.09)	0.396
	True	604	16 (2.6)	588 (97.4)	1.04 (0.98–1.11)	1.03 (0.97–1.08)	0.286
People can reduce their chances of getting the HIV by having just one uninfected sex partner who has no other sex partners	No	81	7 (8.6)	74 (91.4)	1	1	
	Yes	849	24 (2.8)	825 (97.2)	1.06 (0.99–1.13)	1.05 (0.99–1.13)	0.093
There are some special drugs that a doctor or a nurse can give to a woman infected with the HIV/AIDS virus to reduce the risk of transmission to the baby	No	284	18 (6.3)	266 (93.7)	1	1	
	Yes	646	13 (2.0)	633 (98.0)	1.04 (1.01–1.08)	1.03 (1.00–1.07.00.07)	**0.024**

### Predictors of non-disclosure of the HIV status to the most recent sexual partner

After adjusting for age and sex as potential confounders, only marital status was significantly associated with non-disclosure of the HIV status. The prevalence of non-disclosure of the HIV status was 92% (PR:0.08, 95% CI:0.06–0.12, *p* < 0.001) lower among respondents who were married compared to those who had divorced/separated from their most recent sexual partner. Respondents who were widowed/widower to their most recent sexual partner had a 30% (PR:1.30, 95% CI:1.12–1.52, *p* = 0.001) higher prevalence of not disclosing their HIV status compared to those who had divorced/separated from their most recent sexual partner (Table 7).

**Table 7:** Predictors of non-disclosure of the HIV status to the most recent sexual partner among respondents in urban centers in Uganda

Variable	Category	n	Disclosure of the HIV status		Crude prevalence ratio (CI = 95%)	Adjusted prevalence ratio (CI = 95%	P-value
			Yes (*n* = 706)	No (*n* = 193)			
Sex of respondent	Female	664	503 (75.8)	161 (24.2)	1	1	
	Male	235	203 (86.4)	32 (13.6)	0.56 (0.39–0.79)	1.10 (0.80–1.51)	0.519
Age-category (years)	18–24 years	93	66 (71.0)	27 (29.0)	1	1	
	25 and above	806	640 (79.4)	166 (20.6)	0.70 (0.50–1.00.50.00)	0.72 (0.50–1.06)	0.099
Marital status	Divorced/Separated	80	22 (27.5)	58 (72.5)	1	1	
	Married/Cohabiting	703	657 (93.5)	46 (6.5)	0.90 (0.66 − 0.12)	0.08 (0.06–0.12)	*p* < 0.001
	Unmarried/Single	58	23 (39.7)	35 (60.3)	0.83 (0.64–1.06)	0.72 (0.52–1.01)	0.058
	Widow/Widower	58	4 (6.9)	54 (93.1)	1.28 (1.10–1.49)	1.30 (1.12–1.52)	**0.001**
Highest level of education	No formal education	82	54 (65.9)	28 (34.1)	1	1	
	Primary	345	260 (75.4)	85 (24.6)	0.72 (0.50–1.02)	0.90 (0.72–1.13)	0.402
	Secondary	472	392 (83.1)	80 (16.9)	0.49 (0.34–0.71)	0.83 (0.64–1.07)	0.166
Perceived social status	Low	546	414 (75.8)	132 (24.2)	1	1	
	Middle	326	272 (83.4)	54 (16.6)	0.68 (0.51–0.91)	0.97 (0.79–1.20)	0.838
	High	27	20 (74.1)	7 (25.9)	1.07 (0.55–2.06)	1.14 (0.72–1.81)	0.563
Employment status	Employed	850	673 (79.2)	177 (20.8)	1	1	
	Unemployment	49	33 (67.3)	16 (32.7)	1.56 (1.02–2.39)	0.98 (0.72–1.33)	0.918
It is possible to prevent victims of rape from contracting HIV even when raped by an HIV-positive person	Don’t know	79	63 (79.7)	16 (20.3)	1		
	False	232	182 (78.4)	50 (21.6)	1.06 (0.64–1.75)		
	True	588	461 (78.4)	127 (21.6)	1.06 (0.67–1.69)		
People can reduce their chances of getting the HIV by having just one uninfected sex partner who has no other sex partners	No	74	63 (85.1)	11 (14.9)	1	1	
	Yes	825	643 (77.9)	182 (22.1)	1.48 (0.84–2.59)	1.27 (0.86–1.89)	0.225
There are some special drugs that a doctor or a nurse can give to a woman infected with the HIV/AIDS virus to reduce the risk of transmission to the baby	No	266	208 (78.2)	58 (21.8)	1		
	Yes	633	498 (78.7)	135 (21.3)	0.97 (0.74–1.28)		

## Discussion

This study assessed the prevalence and predictors of HIV-related misinformation, HIV testing, and non-disclosure of HIV status among urban dwellers in selected centers of West Nile, Eastern, and Northern Uganda. While almost all respondents reported ever testing for HIV and many had disclosed their status to a partner, the persistence of misinformation remained strikingly high. Education level, duration of residence, HIV testing history, and employment status emerged as significant predictors of misinformation, highlighting how structural and social factors shape knowledge beyond individual service use. Knowledge of special medications to prevent mother-to-child transmission was positively associated with HIV testing, suggesting that concrete, actionable information may motivate uptake of services. In contrast, disclosure practices were strongly influenced by marital status, pointing to the role of relationship dynamics in shaping openness about HIV status. Together, these findings underscore that service availability alone is not enough, programs must also address the social and informational environments that sustain misconceptions and hinder disclosure.

Uganda has made significant progress in expanding HIV testing and treatment services in alignment with the UNAIDS 95-95-95 targets. However, our findings revealed a high prevalence of HIV/AIDS-related misinformation, which may be a significant barrier to achieving these targets. The high prevalence of misinformation reported in our study is indicative of limited access to correct HIV/AIDS-related information among urban dwellers [48, 49]. Although there has been significant investment in HIV/AIDS-related communication for behavioral change interventions, including outreaches [50, 51], many urban dwellers, given their busy and conflicting schedules, did not benefit from them. Interventions aimed at increasing knowledge and awareness of HIV/AIDS are expensive and rarely implemented in most urban settings in Uganda, thereby affecting awareness. Yet, sustained knowledge acquisition depends on constant reminders [52]. The alternative source of information on HIV/AIDS is public healthcare facilities. Yet, some urban dwellers only access them when ill, while others use private healthcare facilities or traditional medicine practitioners who may not have a schedule for health education. Therefore, there is a need for strengthened community-based health education interventions in urban areas to improve access to accurate information, thereby reducing the spread of misinformation. Furthermore, HIV/AIDS programs should consider leveraging digital health interventions as a potential solution to the challenges of urban health communication. Strengthening information dissemination to provide accurate, up-to-date HIV knowledge and counter misinformation is essential for sustaining progress and meeting the 95-95-95 targets. Our study findings, however, don’t concur with those reported in a cross-sectional study conducted in Malawi, where lower levels of HIV-related misconceptions were found [53]. The difference in study findings may partly be attributed to the use of only four misinformation-related questions in the Malawi study compared to the 15-item scale used in our study. This difference in measurement tools highlights the potential influence of question structure and comprehensiveness in estimating misinformation prevalence.

There was no significant difference in HIV-related misinformation across sex and age. The insignificant difference in misinformation is not surprising because all study participants were exposed to similar sources of HIV/AIDS information and accessed services from the same health providers. This uniform exposure likely minimized variation across demographic groups. The study found that nearly two-thirds of the respondents were not willing to disclose the HIV status of a family member. Individuals may not want to disclose their family members’ HIV status as a way of safeguarding them or their households against HIV/AIDS-related stigma and discrimination. There is evidence of people living with HIV/AIDS being discriminated against within families, healthcare facilities, and communities [54, 55]. The proportion that is willing to disclose the HIV status of a family member (61.6%) was lower than what is reported in another low-income country (92.7%) [56].

The prevalence of HIV-related misinformation increased as the level of education decreased. Attending primary and secondary school enhances exposure to HIV-related information. In Uganda, the school curriculum integrates topics on HIV/AIDS transmission and prevention, which helps explain why those who have received some formal education are less likely to be misinformed. Health clubs, such as the Presidential Initiative on AIDS Strategy for Communication to Youth and Straight Talk clubs in schools, contribute to raising awareness about HIV/AIDS [57]. In addition, schools often hold debates, music, dance, and drama competitions that further encourage learners to understand HIV-related information and make informed decisions. Individuals who have attended formal education are also more likely to have been exposed to HIV/AIDS-related information through initiatives by different organisations. The correlation between education level and HIV-related misinformation has also been documented in the literature [58, 59].

Although most studies only describe the relationship between residence (rural vs. urban) and HIV/AIDS-related knowledge [60–63], the current study illustrates that the longer the respondents lived in the study area, the higher their probability of being misinformed about HIV/AIDS. Long-term residents may be consistently exposed to the same sources of information on HIV/AIDS, which may be outdated [64, 65], which can either reinforce accurate knowledge or perpetuate misinformation, depending on the reliability of these sources. In settings where misconceptions about HIV/AIDS are prevalent, long-term exposure to social networks and informal discussions may contribute to misinformation persistence, especially if misinformation is passed down through generations or reinforced by cultural beliefs. Established community-level beliefs and norms about HIV/AIDS may continue to be reinforced over time through local networks, traditional communication methods, or word-of-mouth without the infusion of accurate, evidence-based information. This study highlights the need to consider the duration of residence during HIV programming since it can influence access to accurate information on HIV/AIDS. Future studies should explore the role of community information dynamics in shaping long-term residents’ awareness of HIV/AIDS.

Almost all the respondents have ever tested for HIV, which reflects substantial progress toward the first 95% of the UNAIDS 95-95-95 targets, which aims for 95% of people living with HIV (PLHIV) to know their status [7]. Frequent HIV testing is critical for early detection, timely linkage to care, and prevention of onward transmission. The study also revealed that the majority of those who were positive had been enrolled in ART. This aligns with Uganda’s progress toward the second 95% of the UNAIDS 95-95-95 targets, which aims for 95% of diagnosed individuals to be on ART. ART significantly improves quality of life, reduces morbidity and mortality, and leads to viral load suppression, thereby lowering the likelihood of HIV transmission through sexual intercourse, childbirth, and breastfeeding. Uganda’s “test and treat” policy for HIV/AIDS recommends that all individuals who test positive be enrolled in care, which further explains why almost all the HIV-positive respondents were on ART [66]. However, our findings deviate from those of other scholars, who reported low ART enrolment rates among HIV-positive individuals [67, 68].

While knowledge of special drugs for preventing mother-to-child transmission was positively associated with HIV testing, the borderline statistical significance (lower CI bound at 1.00) suggests that this result should be interpreted with caution. The possible explanation for this is that mothers usually attend antenatal care (ANC) visits at healthcare facilities, where they receive sensitisation about HIV/AIDS and how to reduce the risk of transmission to their babies. These sensitizations often encourage them to undergo HIV testing to know their status. During ANC visits, mothers interact closely with healthcare providers who educate them on how to take their medications if they test positive for HIV. In addition, testimonies from other mothers who know their HIV status and are on medication can motivate women in the community, especially those who suspect they might be HIV positive but have not yet sought medical care. Knowledge of the prevention of mother-to-child transmission of HIV has been reported in the literature as a driver for the uptake of HIV testing [69, 70].

About 1 in 5 respondents who had tested for HIV had never disclosed their status to their sexual partner (s). The fear of stigma and discrimination, violence, and changes in relationship dynamics if one is found to be positive may explain why some respondents had never disclosed their HIV status [71–73]. A lack of knowledge about the benefits of disclosing HIV status and fears of allegations of infidelity or mistrust could further exacerbate non-disclosure. A higher proportion of females compared to males had never disclosed their HIV status to their spouses. Men generally have poorer health-seeking behaviours compared to women and are often reluctant to inquire about the HIV status of their sexual partners, as asking can be perceived as a sign of mistrust [74, 76]. Additionally, the absence of clinical signs and symptoms in females who are HIV positive, along with the fear of violence, loss of economic or family support, and separation from intimate partners, also deters women from disclosing their HIV status [76, 77]. Strengthening community-level interventions to provide accurate, up-to-date HIV knowledge and counter misinformation is essential for sustaining progress and meeting the 95-95-95 targets.

### Strengths and limitations

The study utilises a large sample size, which enhances the generalizability of the findings to urban settings in Uganda. Ngora and Nyero are developing townships that retain some rural characteristics, which may affect the generalizability of the results to all urban centres in Uganda. These areas may not fully represent individuals in larger, more urbanised cities with better access to healthcare information and infrastructure.

The study was prone to recall and social desirability bias since data on HIV-related misinformation, testing, and disclosure were collected through self-reports. Furthermore, our measure of HIV status disclosure was limited to the most recent sexual partner. It did not capture disclosure to multiple partners after HIV testing or the time elapsed since the last test. This limitation could affect the relevance of disclosure as a preventive measure, particularly for individuals who tested negative years ago but have since entered new sexual relationships. Future research should consider examining disclosure patterns across multiple sexual partners and explore the timing of HIV testing to disclosure, offering a more comprehensive understanding of disclosure’s role in HIV prevention. Finally, as the study was cross-sectional, it was not possible to establish causal relationships between HIV misinformation, testing behaviours, and disclosure. We therefore recommend longitudinal studies to explore these relationships further.

## Conclusions

A significant proportion of respondents were misinformed about HIV/AIDS, although most had been tested and disclosed their serostatus to their partners. HIV/AIDS-related misinformation was associated with having a primary education and residing in the area for more than six years. In addition, knowledge of special medications to reduce the risk of transmission to infants was a predictor of HIV testing. Disclosure of HIV status was more common among those who were married or widowed. These findings highlight the need for targeted educational interventions to improve knowledge, increase HIV testing, and support the disclosure of HIV status.

## Data Availability

The data used for this manuscript are available from the corresponding author upon reasonable request.

## Abbreviations

HIV: Human Immunodeficiency virus
AIDS: Acquired Immunodeficiency Syndrome
ANC: Antenatal Care
ART: Antiretroviral Therapy
HCFs: Healthcare facilities
PLHIV: People living with HIV
UNAIDS: The Joint United Nations Programme on HIV/AIDS
UDHS: Uganda Demographic Health Survey
